# Genetics of rotator cuff tears: no association of col5a1 gene in a case-control study

**DOI:** 10.1186/s12881-018-0727-1

**Published:** 2018-12-20

**Authors:** Umile Giuseppe Longo, Katia Margiotti, Stefano Petrillo, Giacomo Rizzello, Caterina Fusilli, Nicola Maffulli, Alessandro De Luca, Vincenzo Denaro

**Affiliations:** 1 Department of Orthopaedics and Trauma Surgery, Campus Bio-Medico University of Rome, Rome, Italy; 2 Molecular Genetics Unit, Casa Sollievo della Sofferenza Hospital, IRCCS, San Giovanni Rotondo, Italy; 3 ALTAMEDICA, Laboratorio Genetica Medica, Roma, Italy; 4 Bioinformatics Unit, Casa Sollievo della Sofferenza Hospital, IRCCS, San Giovanni Rotondo, Italy; 5 Department of Orthopaedics and Traumatology, Azienda Ospedaliera San Giovanni di Dio e Ruggi d’Aragona, University of Salerno, Salerno, Italy; 6 Queen Mary University of London, Barts and the London School of Medicine and Dentistry, Centre for Sports and Exercise Medicine, Centre for Sports and Exercise Medicine, Mile End Hospital, London, UK

**Keywords:** Genetics, Rotator cuff, Tears, Shoulder, Arthroscopy, Gene

## Abstract

**Background:**

The incidence of RC tears increases with aging, affecting approximately 30 to 50% of individuals older than 50 years, and more than 50% of individuals older than 80 years. Intrinsic factors (age or gender), extrinsic factors (sports activity or occupation), and biological factors were identified in the onset and progression of RC tears. The attention in the study of aetiology of RC tendinopathy has shifted to the identification of gene variants. Genes encoding for proteins regulating the concentration of pyrophosphate in the extracellular matrix and genes encoding for fibroblastic growth factors, defensin beta 1 and estrogen-related receptor-beta were analyzed. However, only in one study the role of variants of collagen type V alpha 1 (col5a1) gene in RC tears was assessed. The objective of this study was to determine whether a *col5a1* DNA sequence variant, rs12722 (C/T) was associated with rotator cuff (RC) tears in a case-control study.

**Methods:**

The study included 93 Caucasian patients undergoing surgery for RC tears and 206 patients with no history and sign of RC disease as evaluated by MRI. Patients were divided into two groups. Group 1 included patients with RC tear diagnosed on clinical and imaging grounds and confirmed at the time of surgery. Group 2 (control group) included patients without history or clinical symptoms of RC disorders and with a MRI negative for RC disease. DNA was obtained from approximately 1.2 ml of venous blood using the MagCore extractor system H16 with a MagCore Genomic DNA Large Volume Whole Blood Kit (RBC Bioscience Corp., Taiwan). All study participants were genotyped for SNPs rs12722.

**Results:**

We first estimated that our study had 92% power at *p* < 0.05 to detect a genetic effect size of 2.05 in the RT tears (93 individuals) and healthy population (206 individuals) cohorts, assuming a minor allele frequency for *col5a1* variant rs12722 of 0.5707 in the Italian population (gnomAD frequency). No significant difference in allele and genotype frequencies was observed between RT tears patients and healthy controls. Similarly, no significant association was seen between the RT tears and healthy controls participants in the combined genotype distributions.

**Conclusion:**

In conclusion, no correlations between the SNP rs12722 of *col5a1* gene and RC tears susceptibility was found.

## Background

Rotator cuff (RC) pathologies represent the costliest problem in Workers’ Compensation Systems after low-back pain, with a prevalence rate ranging from 5 to 39% in the general population [1]. The incidence of RC tears increases with aging, affecting approximately 30 to 50% of individuals older than 50 years, and more than 50% of individuals older than 80 years [2].

Although several innovations and valuable surgical techniques were developed in the field of RC treatment, the understanding of aetiology of RC tears and related pathogenesis is still unclear. Intrinsic factors (age or gender), extrinsic factors (sports activity or occupation), and biological factors were identified in the onset and progression of RC tears [1].

However, in the last decade, the attention in the study of aetiology of RC tendinopathy has shifted to the identification of gene variants. Harvie et al. [3] was the first suggesting the genetic susceptibility towards the development of RC tears, demonstrating that siblings had a significantly increased risk of developing RC tears. Furthermore, they demonstrated that RC tears in siblings are more likely to progress over a period of 5 years [4]. Peach et al. [5] found that RC tears were associated with variants of genes (ANHK and TNAP) encoding for proteins regulating the concentration of pyrophosphate in the extracellular matrix (ECM). ANKH is the human homologue of the mouse progressive ankylosis gene (ank) and encodes a multipass transmembrane protein transporting PPi from inside chondrocytes. TNAP encodes the protein Tissue-Nonspecific Alkaline Phosphatase which hydrolyses PPi to Pi and an increase or decrease in concentrations of the enzyme will decrease or increase PPi concentration levels respectively [5]. Moreover, 15 single nucleotide peptide polymorphism (SNPs) were detected in patients with RC tears in the Tenascin-C (TNC) gene [6], and TNC protein is expressed upon tissue damage and has an important role in ECM synthesis and organization in injured tendon tissue. Motta et al. [7] showed a correlation between RC tears and variants of SNPs in genes encoding for fibroblastic growth factors (FGFs) and defensin beta 1 (DEFB1), which are both immune modulating proteins. Furthermore, variants of genes encoding for estrogen-related receptor-beta (ESRRB), which is a crucial protein for the response of tissues to hypoxia, were associated with an increased prevalence of RC tears.

*col5a1* gene encodes for the alpha 1 chain of type V collagen (OMIM 120215), which is a major constituent of bundles in tendons and ligaments [8]. This protein is fundamental in the regulation of the diameter of type I collagen fibrils, and different SNPs of *col5a1* gene were associated with Achilles and elbow tendinopathies [9, 10].

However, only in one study the role of variants of collagen type V alpha 1 (col5a1) gene in RC tears was assessed without any significant result [6]. In this study, in fact, differences in genotype and allele frequencies between patient and control groups did not differ significantly for SNPs in MMP-1, MMP-2, MMP-3, MMP-9, MMP-13, and Col5a1 genes [6].

The objective of this study was to determine whether a *col5a1* DNA sequence variant, rs12722 (C/T) was associated with RC tears in a case-control study.

## Methods

The present study was approved by the local Ethics committee of Campus Bio Medico of Rome. All participants were recruited at the University Hospital Campus Bio-Medico of Rome.

### Study population

All patients are divided into two groups: the first was the study group and the second was the control group. Patients in Group 1 were included in the study if they had RC tear diagnosed on clinical and imaging grounds and confirmed at the time of surgery. Conservative management, including nonsteroidal anti-inflammatory drugs, physiotherapy, and rest, failed in all patients, and they continued to experience unacceptable pain and weakness in the affected shoulder. None of the patients had undergone prior surgery on the affected shoulder. All patients fulfilled the following criteria: (1) positive rotator cuff lag signs on preoperative examination (at least one among Jobe test, Napoleon test, lift-off test, and Patte test), (2) no episodes of shoulder instability, (3) no radiographic sign of fracture of the glenoid or the tuberosities, (4) magnetic resonance imaging (MRI) evidence of cuff tear, (5) RC tear of 1 or more tendons at arthroscopic examination, and (6) no lesion of the glenoid labrum or of the capsule at arthroscopic examination. Patients in Group 2 were included in the study if they had no history or signs of RC tears and if they had an intact RC at MRI.

Patients were excluded in case of primary osteoarthritis of the operated or contralateral shoulder, inflammatory joint disease. Patients in Group 2 were also excluded from the study if they have had history of shoulder pain or rotator cuff pathology diagnosed by imaging or on clinical grounds.

### Genetic analysis

#### DNA extraction

DNA was obtained from approximately 1.2 ml of venous blood using the MagCore extractor system H16 with a MagCore Genomic DNA Large Volume Whole Blood Kit (RBC Bioscience Corp., Taiwan).

#### Genotyping

All study participants were genotyped for SNPs rs12722 by means of ready-to-use TaqMan assay on an HT 7900 platform (Life Technologies, Carlsbad, California, United States of America). The PCR primers and TaqMan probes were designed by Primer Express and optimized according to the manufacturer’s protocol. Genotyping quality was tested by including twelve blinded duplicate samples (four duplicate for each rs12722 SNP genotype: TT, CT, CC) in each 384-well assay. The average agreement rate of duplicate samples was > 99.

### Statistical analysis

Quanto V.1.2.4 was used to determine the statistical power for a given sample size and minor allele frequency [11]. Hardy-Weinberg equilibrium (HWE) for rs12722 SNP in patients and controls was computed through *HardyWeinberg* software (*HardyWeinberg* R-3.3.2 version 1.5.8; http://www.jstatsoft.org). A *stats* R for windows (R stat software package 3.3.2; https://www.r-project.org) was used to compute Odds Ratios and their associated 95% confidence intervals, and *p*-values for the genotypes of rs12722 (C/T). Logistic regression was used for recessive and dominant models.

## Results

The study included 93 Caucasian patients undergoing surgery for RC tears and 206 patients with no history and sign of RC disease as evaluated by magnetic resonance imaging (MRI). Group 1 (study group) included 93 patients with RC tears. 40 (43%) were males and 53 (57%) females, with a mean age of 56.9 + 9.4 (range 40 to 76 years). The dominant arm was affected in 62 (66.6%) patients. Group 2 (control group) included 206 patients without history or clinical symptoms of RC disorders and with a MRI negative for RC disease. This group included 94 males (45%) and 112 (55%) females; mean age: 58.7 + 8.3 (range 41 to 75 years).

We first estimated that our study had 92% power at *p* < 0.05 to detect a genetic effect size of 2.05 in the RT tears (93 individuals) and healthy population (206 individuals) cohorts, assuming a minor allele frequency for col5a1 variant rs12722 of 0.5707 in the Italian population (gnomAD frequency). Genotype data for the rs12722 variant in both study and control groups was tested and found to be in HWE (Table 1). No significant difference in allele and genotype frequencies was observed between RT tears patients and healthy controls (Table 1). Similarly, no significant association was seen between the RT tears and healthy controls participants in the combined genotype distributions (Table 2).

**Table 1:** HWE assessment and case-control association analysis of *COL5A1* rs12722 variant by allele and genotype

	Genotypic frequencies (%)			Allelic frequencies (%)		HWE
	T/T	C/T	C/C	T	C	*p*-value
RC tears patients (*N* = 93)	33 (35.5)	46 (49.5)	14 (15.1)	112 (60.22%)	74 (39.78%)	0.8311
Healthy controls (*N* = 206)	74 (35.9%)	101 (49%)	31 (15.0%)	249 (60.44%)	163 (39.56%)	0.7720

Genotype and allele frequencies are expressed as percentages with numbers (N) in parenthesis. *HWE* Hardy-Weinberg equilibrium

**Table 2:** Case-control association analysis of *COL5A1* rs12722 variant by combined genotype distributions

Genotype comparisons	OR (CI 95%)	*p*-value
TT + CT vs CC	1.0004 (0.504–1.984)	0,9991
TT vs CT + CC	1.0193 (0.611–1.70)	0,9416
TT vs CT	1.0213 (0.596–1.750)	0,9388
TT vs CC	1.0127 (0.477–2.150)	0,9738

*OR* odds ratio, *CI* confidence interval

## Discussion

The main findings of this study is that there is no correlations between the SNP rs12722 of *col5a1* gene and RC tears susceptibility.

s12722 is a common C to T single nucleotide polymorphism (SNP) located in the functional 3′ untranslated region (3′ UTR) col5a1 gene on chromosome 9 at position 134,842,570 [12]. This SNP is considered to be a novel genetic marker for endurance running performance [13] and is also associated with the development of bilateral quadriceps tendon rupture and with chronic Achilles tendinopathy [14].

Following the results reported by Mokone et al. [9] and Altinisik et al. [10] on the correlation between SNPs of *col5a1* gene and Achilles tendinopathy or chronic degenerative tendon changes at the elbow respectively, there has been an increased interest on the potential association of this polymorphism and the risk of developing a RC tear. These results are similar to those reported by Kluger et al. [6] in two different cohorts of patients (1st: 59 patients with RC tears and 32 controls; 2nd: 96 patients with RC tears and 44 controls). However, the authors found 6 SNPs of TNC gene associated with degenerative RC tears, but the most important limitation of their study was that the diagnosis of RC tears was made with ultrasounds, which is an investigator-specific technique with lower sensitivity compared to the techniques (magnetic resonance and arthroscopy) used in the present study [6].

The understanding of the aetiology and pathogenesis of RC tears is still unclear and both intrinsic and extrinsic factors have been advocated [15–26].

In the last decade, a huge number of researchers have focused their attention on the study of genetic susceptibility of RC tears. Peach et al. [5] showed a significant correlation between variants of *ANHK* and *TNAP* genes and RC tears, while Motta et al. [7] found that haplotypes CCTTCCAG in *ESRRB*, CGACG in *FGF3*, CC in *DEFB1*, *FGFR1* rs13317, *FGF10* rs11750845 and rs1011814 were associated with RC tears. Tashjian et al. [27] confirmed the results of Motta et al. [7] on the role of ESRRB in RC tears, showing also that genetic variants of *ESRRB* gene was associated with failure of RC healing, and that positive family history of RC tear increases the risk for healing failure [28, 29].

Our interest in SNP rs12722 of *col5a1* gene raised from the significant correlation between such SNP and Achilles tendon tears or tendinopathy [9]. Mokone et al. [9] enrolled 111 Caucasian patients with Achilles tendon pathology and 129 healthy controls. All patients were genotyped for the BstUI (marker rs12722) and DpnII (marker rs13946) restriction fragment length polymorphisms (RFLPs) within the *col5a1* gene, detecting significant difference in the allele frequencies of the *col5a1* BstUI RFLP between the two groups. They concluded that *col5a1* BstUI RFLP were associated with chronic Achilles tendinopathy [9].

The main strengths of our study are that we have enrolled only Caucasian patients in both study group and control group. Another strength is that the study group included patients who had both MRI and arthroscopic diagnosis of RC tears, while the control group was composed by healthy individuals without history or symptoms of RC disorders who underwent a MRI to confirm that the RC was intact. Furthermore, to improve the quality of the genetic analysis, we have included only Caucasian individuals with Italian origins.

Nevertheless, we are aware of the important limitation of our study. We have tested only the SNP rs12722 of *col5a1* gene and it is possible to hypothesize that genetic variations at this gene could still be present in other SNPs areas and may be related with RC tears. Moreover, we have not collected other parameters such as comorbidities that could be involved as risk factors for the development of RCT. This could be the subject of future endeavours.

## Conclusion

In conclusion, no correlations between the SNP rs12722 of *col5a1* gene and RC tears susceptibility was found.

## Abbreviations

ANHK: human homolog (ANKH) of the mouse progressive ankylosis (ank) gene
DEFB1: defensin beta 1
ESRRB: estrogen-related receptor-beta
FGF: fibroblastic growth factor
HWE: Hardy-Weinberg equilibrium
MRI: magnetic resonance imaging
RC: rotator cuff
RFLP: restriction fragment length polymorphism
ROS: reactive oxygen species
SNP: single nucleotide peptide polymorphism
TNAP: Tissue-nonspecific alkaline phosphatase
TNC: Tenascin-C gene
